# Improving the non-structural preparedness of the selected hospital based on the FOCUS-PDCA^1^ model: action research

**DOI:** 10.1186/s12873-024-01006-w

**Published:** 2024-07-09

**Authors:** Alireza Basiri, Mohsen Abbasi Farajzadeh, Mohammad Belal, Esmail Heidaranlu

**Affiliations:** 1 Health Research Center, Baqiyatullah University of Medical Sciences, Tehran, Iran; 2 Student Research Committee, Baqiyatallah University of Medical Sciences, Tehran, Iran; 3 Nursing Care Research Center, Clinical Science Institute and Nursing Faculty of Baqiyatallah University of Medical Sciences, Tehran, Iran, Baqiyatallah University of Medical Sciences, Tehran, Iran

**Keywords:** Non-structural vulnerability, Safety level assessment, Disasters and emergencies

## Abstract

**Introduction:**

With the intensification of the country’s development process, the expansion of cities and population, and the inclusion of Iran in the accident-prone category, reducing the vulnerability of non-structures has received more attention from the organizations involved. In addition to damage to communities and infrastructure, accidents can affect hospitals and their non-organizational components. Hospitals, as the front line of providing medical services after accidents, must maintain their stability, ensure the safety of their patients and employees, and continue to operate without interruption as in normal conditions. Therefore, it is necessary to evaluate the non-structural safety and their preparedness to ensure they can perform acceptable in critical conditions.

**Methods:**

This applied research was conducted in 2023 (September to December) using the participatory action research method in all selected hospital departments. The level of non-structural preparedness of the hospital was checked using the valid “Hospital Safety Index” questionnaire and the non-structural weaknesses of the hospital were identified. Then, in action research using the FOCUS-PDCA model, a program was implemented to improve the non-structural preparedness of different departments of hospitals in the face of accidents and disasters. The non-structural readiness level of the hospital was compared before and after the implementation of the change.

**Results:**

Based on the evaluation conducted in the present study, the lowest level of safety was observed in the water supply system, office furniture and appliances, and fuel storage. The waste management systems, the fire protection system, and the long-distance communication systems were at a desirable performance level. Although in the evaluation before the change, the overall score of the hospital was 71.01%, and it had a desirable performance level in non-structural factors, in all the involved parts of the hospital, the sensitive, critical, and practical parts in the operation of the hospital had an average and sometimes low safety level. According to the obtained safety score, the safety level of the selected hospital before the change was 7 out of 10 (level seven of safety evaluation = medium). After the change and corrective measures, the non-structural safety assessment score was 76.93, and the hospital’s safety level was raised by one step to 8 out of 10 (8th level of safety assessment = relatively favorable).

**Conclusion:**

The present study showed that the application of Total Quality Management (TQM), primarily its application tool FOCUS-PDCA, is efficient and helpful in improving the non-structural preparedness of hospitals. Using action research in the health field in accidents and disasters can open blind knots in different dimensions of preparedness (structural, non-structural, and functional).

## Introduction

In the last decade, the incidence of unexpected disasters has increased worldwide, with a death rate of about 106,000 people per year [1]. Almost all countries are exposed to unexpected disasters, including hurricanes, floods, earthquakes, fires, droughts, terrorist attacks, volcanic eruptions, chemical accidents, and diseases. Natural disasters can start quickly or slowly and have serious adverse effects on health and social levels and economic consequences [2, 3]. After India, Bangladesh, and China, Iran ranks fourth in unexpected natural disasters [4] So, out of 40 natural disasters reported worldwide, 31 have occurred in Iran, and there is a possibility of their recurrence in the future [5].

If you pay attention to the disaster management cycle and the appropriate prevention and preparedness program, it can be a suitable response when disasters occur and thus reduce the death, injury, disability and burden caused by these disasters [3, 6, 7]. In case of unexpected accidents, hospitals are considered as the most important reception centers for accident victims and because they are among the first organizations involved in the consequences of casualties and injuries of these accidents [8, 9] Hospitals’ preparation as an institution providing healthcare services is essential and critical in reducing deaths and physical injuries caused by accidents, crises, and emergencies [10]. Therefore, it is necessary to determine the level of safety and performance of the hospital against disasters and emergencies [11].

The hospital’s evaluation provides the basis for identifying the components of improving safety and prioritizing interventions according to their type and location to reduce mortality, morbidity, disability, and other social and economic costs [12]. Vulnerability intervention studies usually include in-depth analysis of structural and non-structural risks, health system, and hospital vulnerability. Each of these aspects requires experts with experience in disaster risk reduction. Such investigations typically take several months and may be costly to the hospital [13]. In this regard, the hospital’s safety index is an essential tool to move toward the hospital’s goal, which is to improve the provision of health services to people. These goals include less vulnerability, better preparedness, and safer against disasters and emergencies. In hospitals, whose residents include patients, nurses, doctors, administrative and service personnel, and clients, they should also be responsible for the role of relief when accidents and disasters occur, for this reason, ensuring the stability and efficiency of non-structural components of architecture and facilities. It is necessary [12]. The hospital is made up of many components to provide services to patients, and the relationships between these components must have the necessary coherence so that this institution can perform its duties well Because a defect in any of the components leads to a problem in the process of providing services [14]. One of these components that should be considered is non-structural components. Non-structural components are generally non-structural factors, including architectural, mechanical, telecommunication, electrical, and medical equipment. Non-load-bearing walls, panels, load-bearing walls, suspended ceilings, windows, heating-cooling and ventilation equipment, steam boilers, elevators, emergency power generators, liquid storage tanks, telecommunication devices, and medical equipment and tools are all non-structural elements of the hospital [15, 16].

Architectural components and electrical and mechanical installations in each building include the major part of the construction costs of the entire building. In hospitals, the share of electrical and mechanical facilities is more than in conventional residential, administrative, and commercial buildings. Suppose the cost of providing medical equipment and devices is also considered. In that case, it can be seen that the share of non-structural components of the building in the total investment of a hospital is significant [17, 18].

Past studies show that only 15% of hospital construction costs are allocated to structural elements [19].

85% of all costs are related to architectural, mechanical, electrical, and storage components. Also, hospital equipment and special treatment devices can be added to the previous costs [20].

Comprehensive quality management is among the effective and widely used tools in the field of health that have been used in recent years at the national and even international levels to improve organizational processes. Its important tool is FOCUS-PDCA.

The FOCUS-PDCA tool consists of nine steps, symbolized by the following: (F) Find a problem to improve. Organization (O); turn on (C); Understanding (U); selection (S); plan (P); do (D); Review (C); And, rule (A) - condensed in the acronym, FOCUS-PCDA. The FOCUS-PCDA quality improvement model has been widely used in many fields [21]. is a scientific, coherent, and practical method to improve processes and has a complete and relevant management toolbox to solve organizational challenges with the help of process owners [22].

This study aims to measure the quantitative and qualitative performance of equipment and the vulnerability of non-structural components of selected hospitals in the city of Tehran as a survey, with a special focus on non-governmental hospitals and improving performance with the help of process owners.

## Methods

This applied research was conducted in 2023 (September to December) using the participatory action research method in all selected hospital departments with the participation of the owners of the hospital’s exposure to accidents and disasters in order to improve the hospital’s vulnerability from the perspective of personnel preparation. A statistical sample is a hospital. Field investigations based on the hospital safety assessment tool against disasters, the Persian version of FHSI, include 145 items in 3 general groups: structural safety, non-structural safety, and functional capacity. A statistical sample is a hospital. Field investigations based on the hospital safety assessment tool against disasters, the Persian version of FHSI, include 145 items in 3 general groups: structural safety, non-structural safety, and functional capacity [23]. The data is ranked according to the low, medium and high safety level indicated by the numbers 0, 1 and 2, and the total obtained between 0.0 and 1.0 is classified into 3 levels A, B and C. Be that shows [24].

In this study, non-structural safety, which includes 93 items in the selected hospital, has been investigated. The hospital safety index is a tool designed to evaluate the safety of hospitals and their critical role in responding to disasters and emergencies, and it represents a high quality of care.

**Fig. 1:**
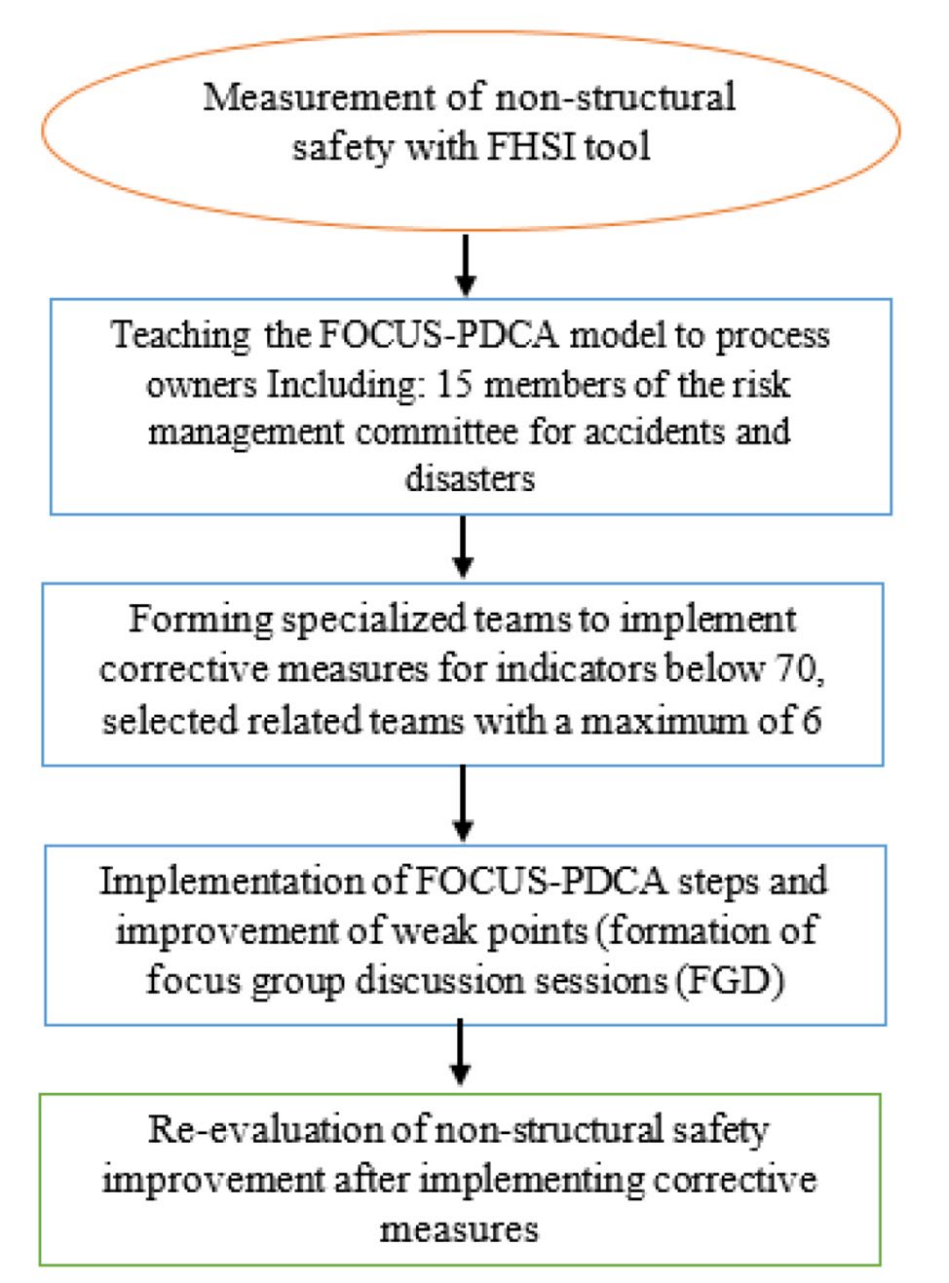
Flowchart of the work execution method

This research is of semi-experimental and interventional (before and after). In this study, non-structural safety, which includes 93 items in the selected hospital, has been investigated. The hospital safety index is a tool designed to evaluate the safety of hospitals and their critical role in responding to disasters and emergencies, and it represents a high quality of care Evaluation team with expert evaluators with experience in hospital construction, providing health services, management, or hospital support activities All specialists involved in the process had the necessary training regarding the objectives and methodology of hospital safety assessment, how to complete the checklist, interpret the results, and prepare the final assessment report Evaluation team with expert evaluators with experience in hospital construction, providing health services, management, or hospital support activities All specialists involved in the process had the necessary training regarding the objectives and methodology of hospital safety assessment, how to complete the checklist, interpret the results, and prepare the final assessment report Organization of the evaluation team Once the desired hospital is selected, the evaluation team includes a hospital manager, treatment manager, medical assistant services manager, health manager, technical and engineering manager, information and communication technology manager, support manager, safety manager, quality improvement manager, manager Nursing, medical equipment manager, structural safety expert, accident and disaster risk management committee secretary, structural safety expert and occupational health expert were formed The characteristics of the hospital and its conditions were investigated [25]. After reviewing the evaluators and the obtained results, continuous meetings were held with the process owners based on the FOCUS-PDCA model, and corrective measures were formulated and implemented considering the three critical indicators of less cost, less time, and high execution capability After the change was made, re-evaluation was done by evaluators regarding the non-structural safety of the hospital (Fig. 1).

### Determining the validity and reliability of the tool

The hospital safety assessment instrument questionnaire by Ardalan et al. has been localized and the Farsi version is called Hospital Safety Assessment Instrument (FHSI). This questionnaire contains 145 items in 3 dimensions of structural, non-structural and functional safety, which have been confirmed in previous studies [23]. This tool is used to evaluate the hospital in three safety levels: weak, moderate and favorable. High safety score or category A (66 − 100%) Medium safety score or category B (36 − 65%) Low safety score or category C (0 − 35%). And based on the three-choice Likert scale according to the Farsi Hospital Safety Index, it was done [26].

### Executive method of the FOCUS-PDCA model

According to the points and standards related to non-structural safety, separately in 12 dimensions of the tool in the items that were at the average, weak, and very weak level (preparation percentage less than 70), FOCUS-PDCA model training in the form of holding a conference, Justification classes and use of visual and audio resources were taught for process owners in 8 two-hour sessions in the conference hall of the hospital. Then, for the continuity and durability of the educational materials, a training booklet designed by the researcher, which has been approved by expert professors in the field of health and disasters and includes general information on non-structural safety and validation items and points that can be modified in the desired items, was distributed among the managers and responsible for the parts involved in the distribution process. Also, three virtual groups were formed on WhatsApp according to the level of the participants to answer their questions and doubts. The reports were followed up through cyberspace. Again, during four weeks, the researcher, together with the hospital risk committee team, visited all the parts of the hospital that were involved in non-structural safety and reminded them of the contents according to their expertise, then with the cooperation of the personnel and officials of each part with Paying attention to their expertise in the desired areas by implementing the FOCUS-PDCA model, a practical solution for correcting the existing weaknesses was implemented by the risk management committee with the help of the process owners of each part, focusing on the non-structural preparedness standard guidelines of the hospital. According to the action research, the leading solutions were focused on the three principles of low cost, low time, and high execution capability. The re-evaluation of the hospital’s non-structural preparedness was done by the researcher and with the help of the managers of the hospital’s risk committee after one month of completing the training. Moreover, finally, the results before and after the change were compared and reported.

## Results

“Hospital Disaster Risk Assessment” tool, non-structural vulnerability in 12 distinct sectors including architectural safety (16 indicators), infrastructure protection, access and physical security (4 indicators), critical systems/electrical systems (10 indicators), long - communication systems range (8 indicators), water supply system (6 indicators), fire protection system (5 indicators), waste management systems (5 indicators), fuel storage systems (such as gas, diesel and diesel) (5 indicators), Heating, ventilation and air conditioning system (HVAC) (8 indicators), office and storage furniture (fixed and mobile) (2 indicators) and medical supplies and equipment used for diagnosis and treatment (19 indicators) (Tables 1 and 2).

**Table 1:** Non-structural safety assessment tool before change

NO	Subject / index	Safety level
		Low = 0 Medium = 1 High = 2
Architectural safety		
1	Major damage and repair of non-structural elements	1
2	Conditions and safety of doors, exits and entrances	1
3	Conditions and safety of windows and shutters	1
4	Conditions and safety of other building facade elements (external walls, facade, etc.)	1
5	Conditions and safety of the roof	2
6	Conditions and safety of fences and walls placed to prevent falling from the roof, bridge, stairs, etc.	2
7	Conditions and safety of surrounding walls and fences	1
8	Conditions and safety of other architectural elements (roof edge wall, plastering of walls, decorations, chimneys and signs)	2
9	Safety of movement outside the hospital building (including the hospital grounds and surroundings)	1
10	Safety of movement inside the building (such as corridors, staircases)	1
11	Safety of internal walls and partitions	1
12	Safety of false ceilings	1
13	Elevator system safety	1
14	Safety of stairs and ramps	1
15	Safety of flooring	2
Infrastructure protection, access and physical security		
16	The location of the places providing services and critical equipment of the hospital according to local risks	2
17	Access ways to the hospital	1
18	Emergency exit and evacuation routes	2
19	Physical security of the building, equipment and patients	2
Critical systems / Electrical systems		
20	Capacity of generators	1
21	Regular evaluation of generators in important areas of the hospital	2
22	Safety of generators	2
23	Safety of electrical equipment, cables and ducts	1
24	Alternative system for local power plant	1
25	Location and safety of control panels and high current disconnect switches and cables	2
26	Lighting system for important areas of the hospital	2
27	Location and safety of internal and external lighting systems	2
28	External electrical systems installed in the hospital premises	2
29	Emergency repair of power generation devices and alternative sources	1
Telecommunications systems		
30	Safety of antennas	2
31	Safety of low and very low voltage systems (Internet, telephone and cables)	2
32	Alternative communication systems	2
33	Condition and safety of cables and long distance communication equipment	2
34	The effect of foreign long-distance communication systems on hospital communication	2
35	Safety of long distance communication systems	2
36	Safety and internal communication systems	2
37	Emergency repair and reconstruction of standard and alternative communication systems	1
Water supply system		
38	Water reserves for hospital operation and services	1
39	Location of water storage tanks	1
40	Safety of water distribution system	1
41	Alternative water source for the main water source	1
42	Complementary pump system	1
43	Emergency repair and reconstruction of water storage systems	1
Fire protection system		
44	The status and safety of the (inactive) fire protection system	2
45	Smoke/fire detection systems (smoke and fire sensitive detectors)	2
46	Fire extinguishing systems (automatic and manual)	2
47	Water storage for fire extinguishing	1
48	Repairs and restoration of fire protection system for critical conditions	2
Waste management systems		
49	Safety of non-hazardous sewage systems	2
50	Safety of liquid waste and sewage (dangerous)	2
51	Solid waste system safety (non-hazardous)	1
52	Solid waste system safety (hazardous)	1
53	Emergency repairs and modifications of all types of hospital waste management systems for critical conditions	2
Fuel storage systems (such as gas, diesel and diesel)		
54	Fuel reserves	2
55	Safety of fuel tanks and cylinders located above the ground	2
56	Safe place to store fuel away from hospital buildings	1
57	Conditions and safety of the fuel distribution system (valves, hoses and connections)	1
58	Reforms and reconstruction of fuel reserves for critical conditions	1
Medical gas systems		
59	Medical gas storage place	1
60	Safety of storage areas of medical gas tanks/cylinders	1
61	Conditions and safety of the medical gas distribution system (for example, valves, pipes and fittings)	2
62	Safety of medical gas cylinders and related equipment in the place of service provision (treatment departments)	1
63	Availability of alternative sources for medical gases	1
64	Maintenance and renewal of medical gas systems in emergency situations	1
Heating, ventilation, and air-conditioning (HVAC) systems		
65	Sufficient space for HVAC equipment	2
66	Safety of HVAC equipment enclosures	2
67	Safety and working conditions of equipment such as steam boilers, exhausts	0
68	Proper support of the channels and checking the flexibility of the channels and pipes that pass through the expansion joints.	1
69	Condition and safety of pipes, fittings and valves	1
70	Conditions and safety of air conditioning equipment	1
71	Operation of air conditioning systems (including areas with negative pressure)	1
72	Emergency maintenance and renewal of HVAC systems	1
Office and storeroom furnishings and equipment (fixed and movable)		
73	Safety of shelves, cupboards and their contents	1
74	Security of computers and printers	1
Medical and laboratory equipment and supplies used for diagnosis and treatment		
75	Safety of medical equipment in operating rooms and recovery rooms	1
76	Safety of radiology and imaging equipment	2
77	Safety of laboratory equipment and supplies	1
78	Safety of medical equipment in the emergency care unit	2
79	Safety of medical equipment in special care units	2
80	Conditions and safety of equipment and furniture in the pharmacy	1
81	Conditions and safety of equipment and tools for sterilization services	1
82	Safety of medical equipment in obstetrics and gynecology emergencies and newborn care	2
83	Safety of medical equipment in burns	---
84	Conditions and safety of equipment in nuclear medicine and radiation therapy departments	---
85	Safety of medical equipment in other services	1
86	Medicines and supplies	1
87	Sterile equipment and other materials	0
88	Dedicated medical equipment used in crises and disasters	0
89	Supply of medical gases	1
90	Mechanical ventilators	1
91	Medical electrical equipment	1
92	Resuscitation equipment (life support)	2
93	Equipment, equipment or emergency trolley for cardiopulmonary arrest	1

**Table 2:** Evaluation of non-structural safety after the change

NO	Subject / index	Safety level
		Low = 0 Medium = 1 High = 2
Architectural safety		
1	Major damage and repair of non-structural elements	1
2	Conditions and safety of doors, exits and entrances	1
3	Conditions and safety of windows and shutters	1
4	Conditions and safety of other building facade elements (external walls, facade, etc.)	1
5	Conditions and safety of the roof	2
6	Conditions and safety of fences and walls placed to prevent falling from the roof, bridge, stairs, etc.	2
7	Conditions and safety of surrounding walls and fences	2
8	Conditions and safety of other architectural elements (roof edge wall, plastering of walls, decorations, chimneys and signs)	2
9	Safety of movement outside the hospital building (including the hospital grounds and surroundings)	1
10	Safety of movement inside the building (such as corridors, staircases)	2
11	Safety of internal walls and partitions	1
12	Safety of false ceilings	1
13	Elevator system safety	1
14	Safety of stairs and ramps	1
15	Safety of flooring	2
Infrastructure protection, access and physical security		
16	The location of the places providing services and critical equipment of the hospital according to local risks	2
17	Access ways to the hospital	1
18	Emergency exit and evacuation routes	2
19	Physical security of the building, equipment and patients	2
Critical systems / Electrical systems		
20	Capacity of generators	1
21	Regular evaluation of generators in important areas of the hospital	2
22	Safety of generators	2
23	Safety of electrical equipment, cables and ducts	2
24	Alternative system for local power plant	1
25	Location and safety of control panels and high current disconnect switches and cables	2
26	Lighting system for important areas of the hospital	2
27	Location and safety of internal and external lighting systems	2
28	External electrical systems installed in the hospital premises	2
29	Emergency repair of power generation devices and alternative sources	1
Telecommunications systems		
30	Safety of antennas	2
31	Safety of low and very low voltage systems (Internet, telephone and cables)	2
32	Alternative communication systems	2
33	Condition and safety of cables and long distance communication equipment	2
34	The effect of foreign long-distance communication systems on hospital communication	2
35	Safety of long distance communication systems	2
36	Safety and internal communication systems	2
37	Emergency repair and reconstruction of standard and alternative communication systems	1
Water supply system		
38	Water reserves for hospital operation and services	1
39	Location of water storage tanks	1
40	Safety of water distribution system	2
41	Alternative water source for the main water source	1
42	Complementary pump system	2
43	Emergency repair and reconstruction of water storage systems	1
Fire protection system		
44	The status and safety of the (inactive) fire protection system	2
45	Smoke/fire detection systems (smoke and fire sensitive detectors)	2
46	Fire extinguishing systems (automatic and manual)	2
47	Water storage for fire extinguishing	1
48	Repairs and restoration of fire protection system for critical conditions	2
Waste management systems		
49	Safety of non-hazardous sewage systems	2
50	Safety of liquid waste and sewage (dangerous)	2
51	Solid waste system safety (non-hazardous)	2
52	Solid waste system safety (hazardous)	2
53	Emergency repairs and modifications of all types of hospital waste management systems for critical conditions	2
Fuel storage systems (such as gas, diesel and diesel)		
54	Fuel reserves	2
55	Safety of fuel tanks and cylinders located above the ground	2
56	Safe place to store fuel away from hospital buildings	1
57	Conditions and safety of the fuel distribution system (valves, hoses and connections)	1
58	Reforms and reconstruction of fuel reserves for critical conditions	1
Medical gas systems		
59	Medical gas storage place	2
60	Safety of storage areas of medical gas tanks/cylinders	1
61	Conditions and safety of the medical gas distribution system (for example, valves, pipes and fittings)	2
62	Safety of medical gas cylinders and related equipment in the place of service provision (treatment departments)	1
63	Availability of alternative sources for medical gases	2
64	Maintenance and renewal of medical gas systems in emergency situations	1
Heating, ventilation, and air-conditioning (HVAC) systems		
65	Sufficient space for HVAC equipment	2
66	Safety of HVAC equipment enclosures	2
67	Safety and working conditions of equipment such as steam boilers, exhausts	1
68	Proper support of the channels and checking the flexibility of the channels and pipes that pass through the expansion joints.	1
69	Condition and safety of pipes, fittings and valves	1
70	Conditions and safety of air conditioning equipment	1
71	Operation of air conditioning systems (including areas with negative pressure)	1
72	Emergency maintenance and renewal of HVAC systems	1
Office and storeroom furnishings and equipment (fixed and movable)		
73	Safety of shelves, cupboards and their contents	2
74	Security of computers and printers	1
Medical and laboratory equipment and supplies used for diagnosis and treatment		
75	Safety of medical equipment in operating rooms and recovery rooms	1
76	Safety of radiology and imaging equipment	2
77	Safety of laboratory equipment and supplies	1
78	Safety of medical equipment in the emergency care unit	2
79	Safety of medical equipment in special care units	2
80	Conditions and safety of equipment and furniture in the pharmacy	1
81	Conditions and safety of equipment and tools for sterilization services	1
82	Safety of medical equipment in obstetrics and gynecology emergencies and newborn care	2
83	Safety of medical equipment in burns	---
84	Conditions and safety of equipment in nuclear medicine and radiation therapy departments	---
85	Safety of medical equipment in other services	1
86	Medicines and supplies	1
87	Sterile equipment and other materials	1
88	Dedicated medical equipment used in crises and disasters	0
89	Supply of medical gases	2
90	Mechanical ventilators	1
91	Medical electrical equipment	1
92	Resuscitation equipment (life support)	2
93	Equipment, equipment or emergency trolley for cardiopulmonary arrest	1

Non-structural safety results in three levels of safety equipment: Low safety: no or zero safety. Moderate Safety: Safety precautions are followed to some extent. And safety, health and safety are very valuable.

This tool evaluates the hospital in three safety levels: weak, moderate and favorable. After analyzing the results in parts with unacceptable safety (percentage less than 70%), some prioritized actions for the lack of safety based on the FOCUS-PDCA (action research) model were reviewed and implemented with the participation of process owners.

**Fig. 2:**
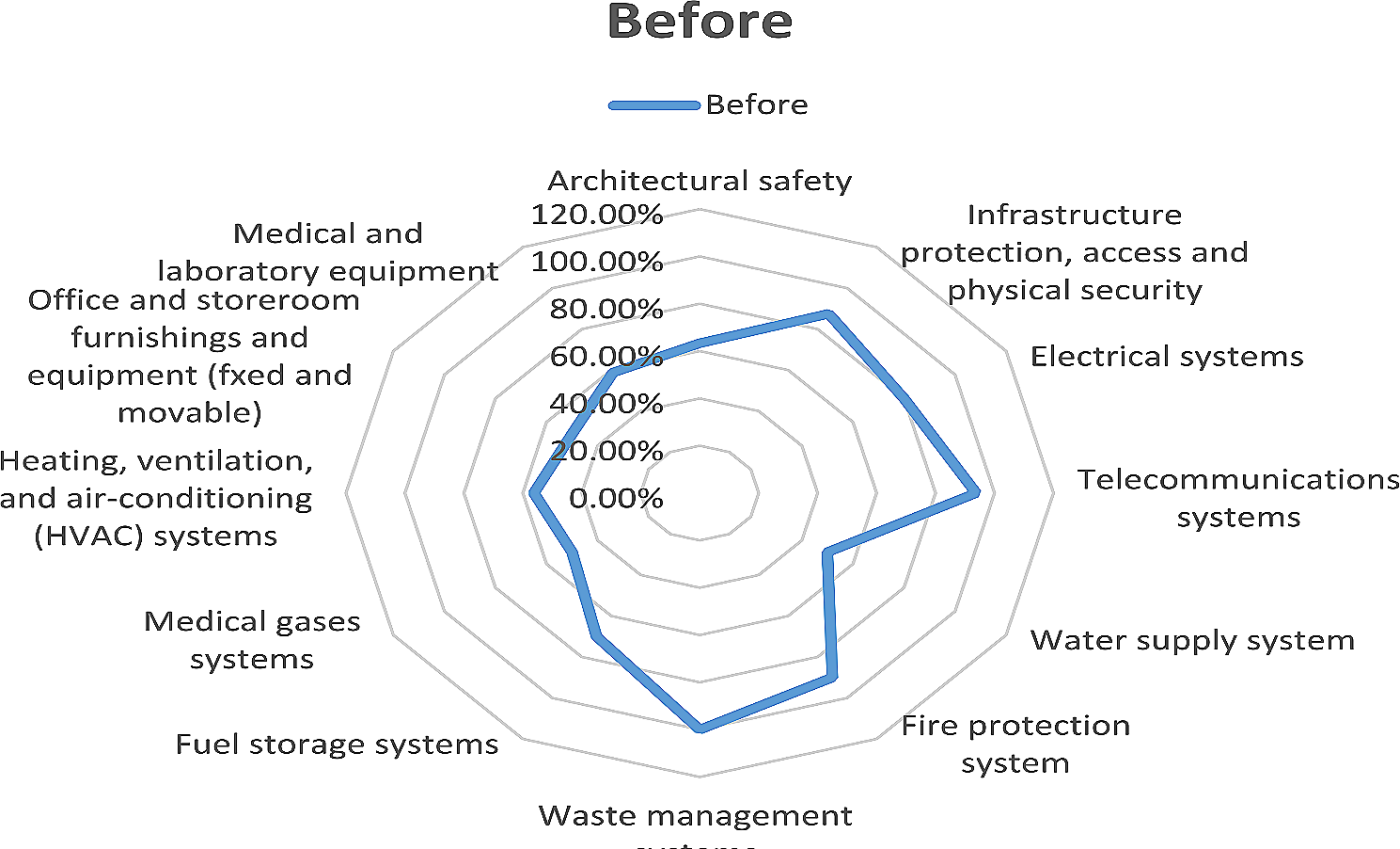
The spider web diagram of non-structural component scores before the intervention

**Fig. 3:**
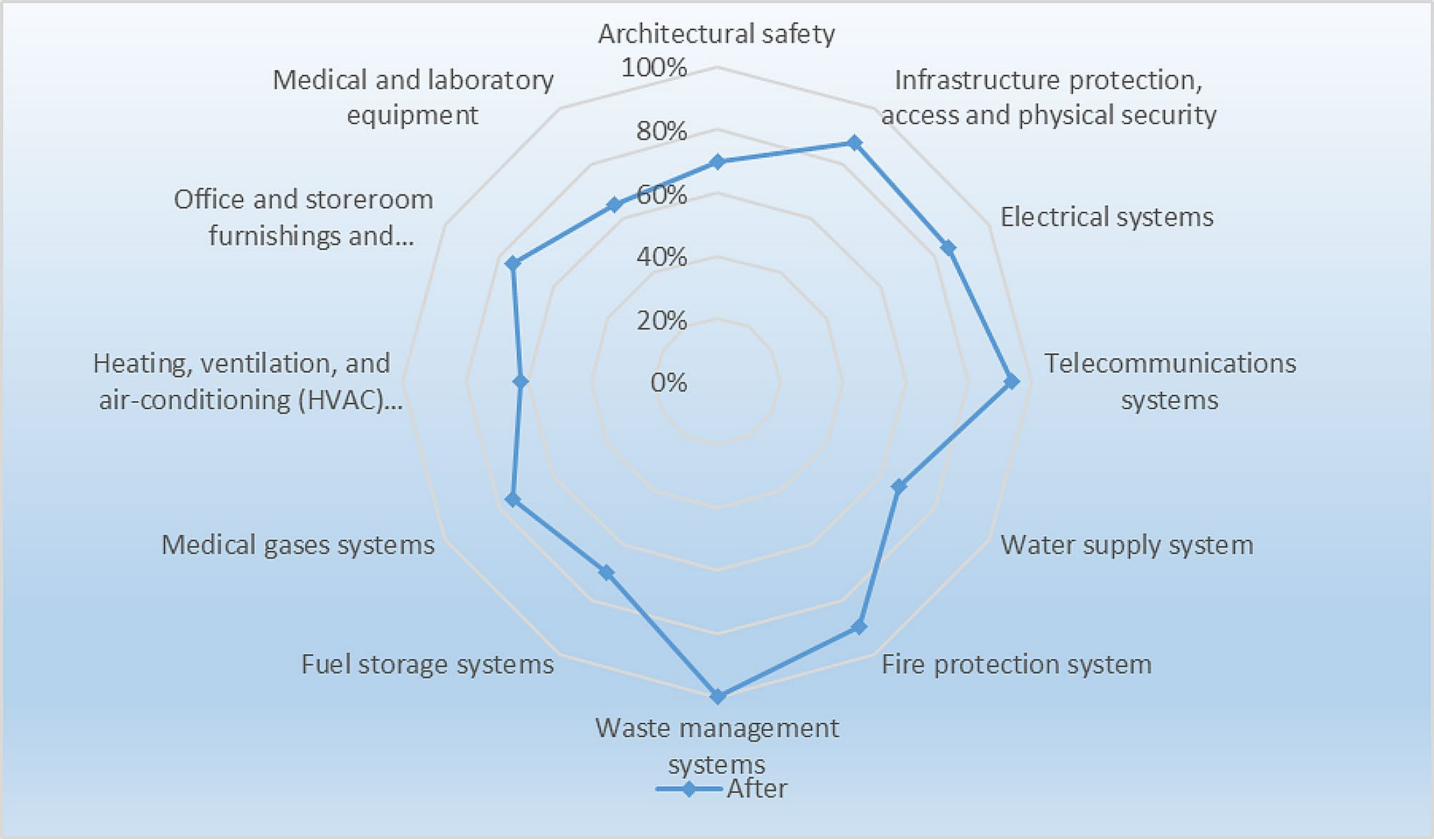
The spider web diagram of non-structural component scores after the intervention

**Fig. 4:**
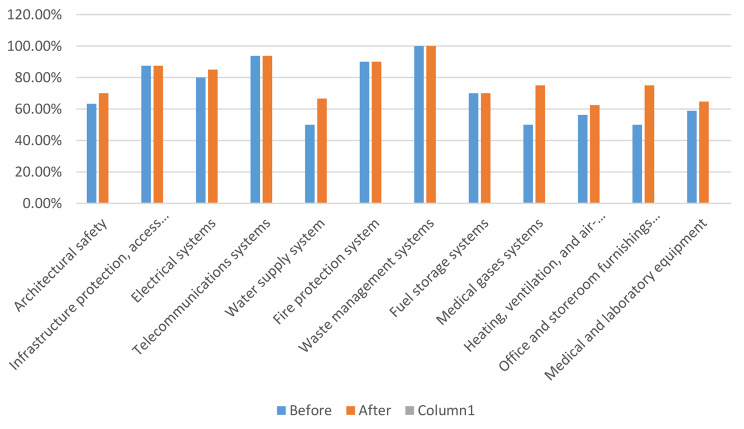
Comparison of improving the safety level of non-structural components before and after the change

Based on the assessment, the lowest level of safety was observed in the water supply system, office furniture and appliances, and fuel storage. In waste management systems, fire protection systems, and long-distance communication systems, they had a desirable performance level (Fig.  2). Although in the pre-intervention evaluation, the overall score of the studied hospital was 71.01% (Table 3), They had a desirable performance level in non-structural factors; In all hospitals, the sensitive, critical and practical departments in the operation of the hospital had an average and sometimes low safety level. According to the obtained safety score, the safety level of the selected hospital before the intervention was 7 out of 10 (level seven of safety evaluation = medium) (Table 4). After the intervention was carried out. Measures were taken based on the evaluation; the lowest level of safety was observed in the heating and cooling system and medical equipment. In waste management systems and long-distance communication systems, they had a more desirable performance level (Fig. 3). Comparison of improvement of safety level of non-structural components before and after change in twelve dimensions. The results showed that the research action and the formation of improvement teams of process owners have played an effective role in achieving non-structural preparation (Fig. 4).

**Table 3:** Average scores based on the evaluation checklist before the intervention

Evaluation of the hospital’s non-structural safety against disasters and emergencies	average score
Safety level of non-structural elements	70.01

**Table 4:** The result of the safety level of the selected hospital based on the evaluation checklist before the intervention

Safety score (maximum)	Safety score (minimum)	Safety class
100	91	**10**
90	81	**9**
80	71	**8**
**70**	**61**	**7**
60	51	**6**
50	41	**5**
40	31	**4**
30	21	**3**
20	11	**2**
10	0	**1**

In the evaluation after the intervention, the non-structural safety evaluation score was improved to 76.93 (Table 5) and the hospital’s safety level was increased by one step to 8 out of 10 (8th level of safety evaluation = relatively favorable) (Table 6). After the implementation of the prioritized corrective measures based on the components, lower cost, less time and more implementation capability (Table 7), the highest level of improvement in medical gas system, office equipment and warehouses 25%, water supply system 17% respectively heating and cooling system 6.75%, architectural safety 6.67%, medical equipment 6.12% and electrical systems 5% (Table 8).

**Table 5:** The average scores based on the evaluation checklist after the intervention

Evaluation of the hospital’s non-structural safety against disasters and emergencies	average score
Safety level of non-structural elements	76.93

**Table 6:** The result of the safety level of the selected hospital based on the evaluation checklist after the change

Safety score (maximum)	Safety score (minimum)	Safety class
100	91	**10**
90	81	**9**
80	71	**8**
70	61	**7**
60	51	**6**
50	41	**5**
40	31	**4**
30	21	**3**
20	11	**2**
10	0	**1**

**Table 7:** Extracted priorities for change after implementing the FOCUS-PDCA model

NO	Area	Improvements in areas that need to be changed after the implementation of FOCUS-PDCA
1	Architectural safety	✓ Installation of guards and fences in the walls ✓ Installation of roof guards and fences ✓ Installation of stairs ✓ Installation of stair railings
2	water supply	✓ Locking the entrance doors of tankers ✓ Lack of access to the place of storage of tankers ✓ Use of supplementary and backup pumps
3	Fuel storage systems	✓ Change in storage location ✓ Increasing the amount of stored fuel ✓ Installation of flexible connections for pipes
4	Medical gases systems	✓ Moving the place of production and storage of medical gases and oxygen generators ✓ Using alternative sources for medical gases
5	Heating and cooling(HVAC)	✓ Clamping the connection of pipes ✓ Bracing of cables connected to equipment ✓ Use of flexible connections
6	Office and storeroom furnishings and equipment (fixed and movable)	✓ Installation of braces ✓ Use of screw packages ✓ Locking the cupboards ✓ Strapping of cages and computer equipment
7	Medical and laboratory equipment	✓ Installing a sterile device (CSSD) in the operating room department ✓ Increasing the required oxygen production capacity

**Table 8:** Percentage of improvement of safety of non-structural components after change

NO	Title	Before the intervention (%)	intervention dimension (%)	Promotion rate (%)
1	Architectural safety	63.33	70	6.67
2	Infrastructure protection, access and physical security	88	88	---
3	Electrical systems	80	85	5
4	Telecommunications systems	93.75	93.75	---
5	Water supply system	50	66.66	16.66
6	Fire protection system	90	90	---
7	Waste management systems	100	100	---
8	Fuel storage systems	70	70	---
9	Medical gases systems	50	75	25
10	Heating, ventilation, and air-conditioning (HVAC) systems	56.25	63	6.75
11	Office and storeroom furnishings and equipment (fixed and movable)	50	75	25
12	Medical and laboratory equipment	58.82	6	6.18

## Discussion

In the field of national crisis management, the United States Department of National Security considers disaster preparedness as one of the stages of crisis management, which includes data collection, research, planning, organizational development, provision of resources, training, obtaining documents and certificates, equipment, and records management [27]. Also, WHO has identified the lack of training for preparedness against unexpected events and disasters at the state level and ordinary people as one of the main reasons for the high losses caused by natural disasters [28]. In the studied hospital, the condition of non-structural factors was average. The findings of this study showed that the overall average non-structural safety of the studied hospital after the implementation of the FOCUS-PDCA model and the participation of process owners in each area in multiple group discussion sessions was reported to be 76.93%, which is evaluated as (good) according to WHO standards. The study by Dargahi A. et al. (2017) showed that the average non-structural vulnerability to disasters is 41.5%, and the average preparedness of healthcare centers in Coping with disasters was 18.3% [13]. In addition, Arab M et al. (2009), in a study titled A study concluded from their study in Tehran hospitals, that The operational preparedness of hospitals in response to disasters is 51%. Both studies recorded a lower percentage of preparedness than the present study [29]. However, Ojaghi S et al. (2009), in a study found that the preparation of teaching hospitals to deal with disasters could be more robust [30]. Based on the study by Al Khalaileh MA et al. (2012), 65% of nurses stated that hospitals’ preparedness level to deal with disasters could be much higher. The study showed that Jordanian hospitals were poorly prepared against disasters [31]. In the study of Hatami H and colleagues (2017) entitled Functional, structural, and Non-structural Preparedness of Ahvaz Health Centers against Disasters, they obtained a safety index of 4 out of 10 and a non-structural safety of 54.82% [15]. In the study of Sabzghabaie A. et al. (2013) titled Hospital Safety in Hospitals Affiliated with Shahid Beheshti University, 49.44% of disaster preparedness was evaluated for Shahid Beheshti Hospital, which did not match the results of our study [32]. In a study conducted by Arab M and colleagues (2009), their hospital’s preparedness against earthquake events and their relationships at public hospitals showed that in 47% of the studied hospitals in the area, The safety of dangerous equipment and materials against accidents was at the medium level of preparedness and 14% were at the low level of preparedness [29]. In addition, in the study of Jagnarine S, which was conducted on 45 hospitals in the Caribbean, under the title Safe hospitals in the Caribbean, only 2% of hospitals had relatively good safety, 80% had moderate safety, and 18% had low safety, which The results of our study were different [33]. The difference in the preparedness of the non-structural components of hospitals is due to several factors, including the difference in the research environment, data collection method, oral interview, written questionnaire or observation, when collecting data, training, and expertise of the people who collected the data, the type of checklists used. Moreover, relatively long-term studies are concerned.

In the Abbasabadi-Arab et al. (2023) study done in 604 hospitals, the average non-structural score was 65.24, and functional safety was 63.36. The provinces of Hamedan (76.81 and Kerman) had the highest score, and the provinces of Yazd (53.74) and Lorestan (57.31) had the lowest score [34]. The study by Asadi G. and colleagues (2022) titled Assessment of Safety Status and Functional, structural and Non-structural Preparedness of Health Centers in Hamadan against Disasters was done. The results of preparation level and non-structural safety level are 63.96%. The level of non-structural safety is at an acceptable level that can be improved [3]. Based on Takzadeh H and colleagues’ (2015) study on the safety Assessment of urban and rural health service centers. Isfahan health homes and health centers in barriers and emergencies, the average scores of non-structural factors in urban and rural centers were 74.2 and 82.5, respectively [35].

In the study of Tabrizi et al. in East Azarbaijan, the safety rate of non-comedogenic drugs was reported to be 61.75% [36]. The study conducted by Amerion A and colleagues (2010) showed that the preparedness of hospitals to deal with disasters is more than 70% [37]. Mahboobi et al.‘s study (2008) reported that the average preparedness of three hospitals and healthcare centers in Kermanshah regarding equipment and facilities was 74.6%, compared to The present study, which is almost equal [38]. In a study conducted by Asefzadeh S et al. (2016). The preparedness of the studied hospitals in dealing with disasters with an average non-structural safety of 67.71% (safety average) in two hospitals [39]. In a study by Cliff BJ et al. in a rural hospital in the United States, the preparedness of these hospitals was evaluated at 78% [40, 41]. In the study by Zhong S et al. (2014), the preparedness of hospitals was 81% [42]. The results of the present study are consistent with other studies that examine the level of preparedness of hospitals in Tehran against disasters, and it confirms that the non-structural performance of hospitals in accidents and disasters is at an average to a high level.

However, it should be noted that according to the weak chain theory, the non-structural elements of a hospital are like the links of a chain, and a weakness in any of them may cause the whole complex to break. Therefore, the average values of the safety level of the set of non-structural factors of a hospital cannot be a suitable indicator for the level of vulnerability of a hospital. Therefore, investigating each non-structural factor and predicting solutions to improve the functional level and implement their corrective measures will be closer to reality. The results of our study showed that the non-structural safety in the hospital under our study was 70.01% before and after the intervention. They were increased to 76.93%, which was a good-high level.

### Architectural safety

The hospital’s non-structural safety percentage in architecture before the change and implementation of the FOCUS-PDCA model by the process owners was 63.33%. These components do not have a direct effect on the operation of the hospital. However, their destruction during accidents can seriously disrupt the efficiency of different hospital departments. False ceilings, facades of internal walls and columns, plasterwork, and non-load-bearing separating blades are highly vulnerable if they are not well connected to the main structure of the building. After implementing the FOCUS-PDCA model and applying corrective measures in the areas of the safety of the surrounding walls and the safety of movement inside the building (such as corridors and staircases), such as installing guards and fences on the walls, roofs, installing stair railings, installing stair railings, etc., the level score Architectural safety was increased to 70%, in a study by Luke J. et al. (2023) the preparedness and evaluation of 6 tertiary and rural hospitals in Australia found that the architecture’s safety components are very resistant to disasters, and each recorded high HSI scores. Differences in structure, architectural safety, and continuity of supply of critical services have been identified. The safety of the architectural components of the studied hospitals has a score of 79% [43]. In a study conducted by Lestri F et al. (2022), the safety score of architectural components in the studied hospitals located in four provinces of Indonesia, namely in Jakarta, Yogyakarta, North Sumatra, and West Java, the percentage is between 36% and 65% [44]. In a study conducted by Asefzadeh S et al. (2016), the safety score of architectural components in Velayat Hospital was 88.88%, and Rajaee Hospital was 69.44% [39]. In the study by Sadeghi et al. (2015), suitable Solutions and Missions in These Situations in the Second Semester of 2015 were conducted regarding the safety level of the architectural components of Baath Hospital. 100%, family hospital 58% and Ahvaz 578 hospital 41% had safety [45], which is different from the results obtained in the present study, which shows that the level of activity in order to obtain the necessary safety according to the standard in these centers is different. have been.

### Infrastructure protection, access and of physical security

88% of the studied hospitals had good Infrastructure protection, access, and physical security resources in the emergency exit system. The emergency exit routes, roads, and stairs needed to be marked on most hospital floors. In this regard, it is recommended to take some measures to correctly mark emergency exits, train staff to guide people and move supplies and equipment that make the use of emergency exits difficult. In the study by Yang et al. (2023) regarding assessing the resilience of critical infrastructures in France, which has been done in two ways, the safety level of infrastructure protection and the availability of physical resources at an acceptable level existed [46, 47]. In the study of Suparni, the current safety levels of the hospital are such that patients, hospital staff, and the hospital’s ability to function during and after an incident are potentially at risk [48]. In the study of Amerion A et al. (2010) regarding the safety level of infrastructure protection and access to physical resources in the emergency exit system, a score was obtained (72-100%), which is close to the score obtained in the present study [37]. In Hatami H et al.‘s study (2017), evacuation or emergency exit has the lowest score, with 33.3% [15]. preparedness for confronting crises such as floods, earthquakes, fires, and storms in some selected hospitals in Iran. Iranian Journal of Military Medicine preparedness rate (48.8%) has been obtained [49], and the study of Hojat M et al. (2012) reported the preparedness rate (39.63%) And the study of Kavari SH and colleagues (2006) [50]. to Shiraz University of Medical Sciences. Health Information Management analyzed the preparation level of this department, which is at 50%, which is low [51]. In the study of Zaboli R and colleagues (201), the level of preparedness (31.6%) was reported [52]. In the study of Hosseini Shokouh S and colleagues (2008), the level of preparedness in infrastructure is reported as 33.3% [53]. In the study of Mohammadi Yegane S et al., the level of preparedness regarding emergency exits is reported as 14% [54] and the review of the conducted studies is in line with the present study.

### Critical systems/electrical systems

In the investigation of the hospital under study, the safety of the hospital in critical systems/electrical systems was reported as 80%. In order to improve the safety level of the electrical sector, it is recommended that the main cables located in the channels are installed in such a way that their standard and safety distance from each other is observed so that in case of an accident in one of the cables, the other cables will not suffer an accident. It was also recommended to consider an emergency lighting system using rechargeable generators (batteries) for power outages so that emergency evacuation is not associated with casualties. In addition, an uninterrupted power supply (UPS) should be provided for critical parts of the hospital during an earthquake. Considering that in this area, the promotion team was not formed due to a score above 70%, but the ongoing and routine actions of the hospital’s engineering department in the areas of critical systems/systems for maintaining the standard distance of cables from each other, as well as the placement of cables in safe channels and ducts, practically caused in the following evaluation, the safety level was increased to 85%. In the Tal MA (2021) et al. study, acceptable accuracy was obtained in assessing the current situation and predicting the future situation [55]. In the study of Zegarra RO (2023), prehospital and disaster medicine was conducted in 18 government hospitals. The average safety score of the critical systems of the studied hospitals is 63% [56]. In the study of Kjølle et al. (2012). Reliability Engineering and System Safety have been done. Regarding the risk analysis of critical infrastructures emphasizing electricity supply and its interdependencies in the hospitals of Oslo, Norway, a severe power cut can have cascading effects and consequences for other infrastructures [57]. In a study by Asefzadeh et al. (2016), the safety score of electrical systems in Velayat Hospital is 81.25%, and Rajaei Hospital is 43.75% [39]. In the study by Mohammadi Yegane et al. on the safety of electrical systems, Golestan Hospital, with 100%, and Family Hospital, with 43%, had the lowest percentage of safety in electrical systems [54]. The scores obtained in the studies conducted in Tehran’s Velayat and Shariati hospitals are almost equal.

### Telecommunication systems

The hospital obtained 93.75% non-structural safety in telecommunication systems in the current study. It must be accepted that admitting the wounded is highly dependent on establishing external communications and coordinating within the hospital; there is an urgent need to establish internal communications. In this regard, strengthening and restraining equipment and cables in the hospital’s communication system, creating multiple layers of secure communication, and using wireless technology can guarantee communication during incidents. The study by Bisanda MP et al. (2023) showed that the resilience of mobile and sustainable telecommunications communication systems in disaster conditions is critical and important [58]. It was done in the study of Lamine H et al. (2022). The evaluation of the level of preparedness and response, including nine hospitals of the University of Tunis in disasters, showed that 7 of the nine university hospitals had safety category “B” in communication systems with overall safety indicators between 37% and 62% [59]. The study by Zaboli R et al. (2014) conducted a study on communication and information systems related to emergency management, which stated that the personal information of personnel is the most important and critical element for any system, especially crisis management. In a disaster, key personnel information must be available for quick response. In this study, the preparedness of communication and early warning was reported as 46.2%, which was lower than the average [52].

On the other hand, in the study of Amerion A et al. (2010), the percentage of preparedness in this area was reported as 66% [29]. In a study conducted by Asefzadeh S and colleagues (2016), the safety score of communication systems in Velayat Hospital and Rajaee Hospital was 92.85% and 42.85% [37]. In the study of Sadeghi M et al. (2015), hospital 501, at 81%, and Hospital 578 in Ahvaz, at 26%, had the highest and lowest percentage of safety in communication systems, respectively [45].

### Water supply systems

The studied hospital obtained a 50% safety level in water supply systems. Failure to supply drinking water required in emergencies for at least 72 h, considering the number of approved beds in the hospital and its occupancy rate, can cause the hospital to face a severe challenge at the time of response and water cutoff. Although most hospitals are usually equipped with an emergency water supply system, these systems are primarily designed for normal conditions and cannot respond in emergencies. After the implementation of the FOCUS-PDCA model by the process owners and the implementation of corrective measures in the field of the water supply system, such as locking the entrance doors of tankers and no access to the place of storage of tankers and the use of supplementary and backup pumps, the safety level score was increased to 66.66%. In the study of J EL-Matury H et al., the safety level of this system has been evaluated as “B,” which shows their ability to function during and after disasters.

Moreover, disasters are potentially at risk [60]. In a study by Luke J et al., twenty-two articles were reviewed using hospital resilience checklists and assessing non-structural components and water supply systems against natural disasters in ten countries. It shows that the average Amagi score of water systems in the reviewed studies is around 46%, which requires immediate intervention and the removal of relevant deficiencies [61]. In the study of Asadi G. et al. (2022), the lowest level of preparedness in the non-structural field in Hamedan health centers is reported in water supply systems at 38.78% [4]. In the study of Salari H and colleagues (2009), the most preparedness related to the water supply system was stated with 85% of the preparedness rate [28]. In a study conducted by Asefzadeh S. et al. (2016), the safety score of water supply systems in Velayat Hospital is 100%, and Rajaei Hospital is 70% [39]. In the study of Sadeghi M et al. (2015), especially in the water supply systems of Golestan Hospital with 84% and Family Hospital with 24%, they had the lowest percentage of safety in water supply systems [45].

### Fire protection system

The studied hospital achieved a 90% safety level in fire protection systems. The safety status of this area was satisfactory in all parts of the hospital under study. Most of the hospitals had fire alarm systems and manual fire extinguishers. However, after an earthquake, it may not be possible for the fire department to be present at the hospital in time, in which case the fire may spread and spread. One of the measures that should be considered is using automatic fire extinguishing systems in areas such as warehouses, parking lots, and areas where automatic fire extinguishing does not harm the existing equipment and employees. In the study by Irwanto et al. (2021) conducted in a hospital in Andozi, the safety level of the fire protection system was reported to be 83% [62]. In the study of F Lestari et al. (2022), hospital safety index guidelines include four parameters: types of risks, structural safety, non-structural safety, disasters, and emergency management. The overall safety index is 67% and at level A, which means the hospital will likely maintain its performance in emergencies and disasters. Based on the evaluation, the fire protection system’s average safety score in DKI Jakarta’s provinces is 76%, Yogyakarta is 70%, and West Java is 66% [63]. In the study of Asadi G et al. (2022fire extinguishing systems are reported with 74.37% [4]. The study by Hatami H and colleagues titled Functional, structural, and Non-structural Preparedness of Ahvaz Health Centers against Disasters was done. Although most of the studied health centers had a sufficient fire safety level of about 72.45%, they did not have any information about fire detection and control systems. Despite the presence of fire extinguishers in most centers, they lacked a fire alarm system [19]. In the study of Mohammadi et al. (2011), fire control preparedness was reported to be 57% [54].

### Waste management systems

In the field of waste management systems, the studied hospital obtained a 100% safety level. In the systematic waste management of healthcare centers, it is practically considered to implement a fixed and continuous program to remove the storage and collect production waste inside and transport it outside the sources of production of such waste. Therefore, hospital waste collection and transportation management are placed in two separate departments. Collection and transportation inside the hospital and collection and transportation outside the hospital. All hospitals have places for temporary storage of hospital waste, and some are equipped with a waste incinerator system.

However, it should be noted that in emergencies, in addition to the volume of patients to medical centers and, as a result, the production of hospital waste will increase, the planned structures for separation, burning, or transfer of waste outside the hospital will also be disrupted. In Blanchette RA et al.‘s (2023) study, in the field of waste management systems, the safety score is reported between 33% and 97% [64]. In Awodele O et al.‘s (2016) study, all facilities have identical waste management processes, including segregation, on-site collection/transportation, on-site storage, and off-site transportation. Medical waste collection staff mainly use gloves as personal protective equipment. Intervention programs helped ensure compliance and safety of processes. Only Hospital B provided on-site treatment of its waste (sharps only) with an incinerator [65, 66].

### Fuel storage system

In the fuel storage systems (such as gas, diesel, and diesel), the studied hospital achieved a 70% safety level. Considering that the hospital’s score in this area was good, corrective measures were suggested, and the process improvement was not accepted. Suggested corrective measures in this area include a change in the place of storage of stored fuel, as well as the way of storing fuel and installing flexible connections for pipes in the place of connection to the tanks, which can help in improving the safety of the fuel storage system. In the study of Muhammad-Idris et al. (2022), epidemics, riots, fires, and gas explosions, among other things. Serious effects have been reported in 65 to 85% [67]. In the study of Vichova K et al. (2019), an assessment was made to provide emergency fuel sources to hospitals. The evaluated hospitals are divided into types of faculty hospitals, regional hospitals, city hospitals, and private hospitals. Zlín Hospital is a type of private hospital that was evaluated in the first stage, and the level of fuel storage system preparedness in this hospital is 57.14%; the second stage is the evaluated hospital from the Czech region, which is a regional hospital and the level of preparedness is 33.34% in the field of energy supply. An emergency has been reported. In the third stage, the hospital in the South Moravia region, which is a teaching hospital, was investigated, and the level of preparedness of this hospital was 94.45% in the field of fuel systems in the region. In the fourth stage, a hospital from the Hradec Králové region, which is a type of region, is evaluated. It has 71.43% preparedness in the field of emergency fuel supply.

Finally, the hospital from the Ústí nad Labem region, which is a type of urban hospital, was rejected, and this hospital received 64.29% of the preparedness score [68]. In a study conducted by Asefzadeh S et al. (2016), the safety score of fuel storage systems in Velayat Hospital is 87.50% and Rajaei Hospital is 50% [39]. In the study of Sadeghi M et al. (2015), especially in fuel storage systems, Golestan and Family Hospital had the highest safety percentage with 90%, and Ahvaz 578 Hospital had the lowest safety percentage with 49% [45]. Regarding the preparation of non-structural components in the safety of heating, cooling, and air conditioning systems, 84% of the reviewed studies had the highest and 45% the lowest percentage of safety in the medical gas system [69]. In a study conducted by Asefzadeh S (2016) et al., the safety score of cooling, heating, and air conditioning systems in Velayat Hospital was 85.71% and 42.85% in Rajaei Hospital [39]. In Sadeghi M et al. (2015) study on the safety of heating, cooling, and air conditioning systems, 501 hospitals had the highest safety percentage at 77%, and family hospitals had the lowest safety percentage at 20% [45].

### Medical gas system

In the field of medical gas systems, the studied hospital obtained a 50% safety level. The fall of the equipment and facilities of the adjacent building on the medical gas building can cause irreparable critical and material damages. In some hospitals, a central oxygen system is likely to lose its efficiency during an earthquake. Therefore, portable medical gas systems should be considered in all departments for emergencies. After the implementation of the FOCUS-PDCA model by the process owners and the implementation of corrective measures in the field of safety of the medical gas system and the relocation of the place of production and storage of medical gases and oxygen generators and the use of alternative sources for medical gases as well as the use of valves equipped with seismic cut-off for the oxygen system The center that will prevent gas wastage and the consequences of its release during the crisis, the safety level score in this area was raised to 75%. In Goniewicz et al.‘s (2023) study, the findings have been interpreted cautiously, and the most preparedness and performance related to the index of medical gases has been reported [70]. A study by Raju R et al. (2019) in India, this study concluded that determining an additional area for storing cylinders, adding another oxygen storage tank, and accuracy A lot in planning, design, and operation can increase the level of preparedness for medical gases in disasters [71]. The study by Gómez-Chaparro et al. (2018), consisting of 12 Spanish hospitals with land area and number of beds between 2314 and 23,300 square meters and 20 and 194 square meters, reports that the average annual consumption rates of medical gases by area, number of beds, number of inpatients, outpatients; The number of endoscopies, the number of emergency interventions, the number of hospital surgeries on another note, and the number of hospital discharges should be considered as suitable variables for quantification, measurement and evaluation. In evaluating the safety level of medical gas systems in the studied hospital, an average preparedness score of 68.35 [72]. was reported. In a study by Asefzadeh et al., the safety score of the medical gas system in Velayat Hospital was 57.14%, and Rajaee Hospital was 50% [39]. In Sadeghi et al. (2015) study, Golestan Hospital had the highest safety percentage at 89%, and Ahvaz 578 Hospital, with 20%, had the lowest safety percentage of medical gas system [45].

### Heating, ventilation and air conditioning (HVAC) systems

In the field of Heating, ventilation, and air conditioning (HVAC) systems in particular departments, the studied hospital obtained a 56.25% safety level. These systems are made up of many different components, which are different in each hospital, and there is a possibility of damage and disturbance in each of them, even in normal conditions. One of the sectors that can be expected to be damaged during natural disasters is the heating-cooling and air-conditioning systems in particular sectors. All the equipment of these systems must be braced, and in the place of connecting the pipes and cables to the equipment, flexible joints that can withstand seismic conditions should be used. After the implementation of the FOCUS-PDCA model by the process owners and the implementation of corrective measures in this area, including measures such as bracing of pipe connections, bracing of cables connected to equipment, and the use of flexible connections, the safety level score was increased to 63%. Eight articles were examined in the study by Chair SY et al. (2023) in China under the title of heating, cooling, and air conditioning systems in hospitals. The findings of this review show the range, similarities, and differences in the performance of HVAC systems in different countries. In the early stages of the outbreak of COVID-19, various researchers reported that the main route of transmission of COVID-19 is through respiratory droplets. Recently, scientific evidence suggests that it is transmitted through the air. COVID-19 is highly transmissible in poorly ventilated and closed environments, and heating, cooling, and air conditioning systems have not performed well in the studied studies [73]. In the study of Moradi SM et al. (2021), 24 studies have been examined. The essential findings of this study were categorized into five main categories: risk analysis method, type of disaster, hospital safety methods, hospital components, and key outcomes of risk analysis and hospital safety assessment.

Regarding the preparation of non-structural components in the safety of heating, cooling, and air conditioning systems, 84% of the reviewed studies had the highest and 45% the lowest percentage of safety in the medical gas system [69]. In a study conducted by Asefzadeh S (2016) et al., the safety score of cooling, heating, and air conditioning systems in Velayat Hospital was 85.71% and 42.85% in Rajaei Hospital [39]. In Sadeghi M et al. (2015) study on the safety of heating, cooling, and air conditioning systems, 501 hospitals had the highest safety percentage at 77%, and family hospitals had the lowest safety percentage at 20% [45].

### Ofce and storeroom furnishings and equipment (fxed and movable)

In Office and storeroom furnishings and equipment (fixed and movable), the studied hospital obtained a 50% safety level. It is necessary to use various fixing methods in the equipment and furniture department, including installing braces, screw packages, locking closets, and strapping cabinets and computer equipment. After the implementation of the FOCUS-PDCA model by the owners of the process and the implementation of corrective measures in this area, including measures such as: installing braces, using screw fasteners, locking closets, strapping cages, and computer equipment, the safety level score was increased to 75%. In terms of equipment, all the studied office centers, such as shelves, computers, and office furniture, needed to be in better condition. In the event of an accident, they could be turned off completely. The shipping investigation process is done in the study of Ratwani RM et al. (2023) in the state of Pennsylvania, USA. Out of 450 reviewed reports, the most frequent was in office supplies and furniture and storage (fixed and movable). The safety problem related to the types of fixing methods, including the installation of tie-downs, screw packages 33.34%, strapping of cages and computer equipment 28.2%, and locking 28.2% have been reported [74]. The study by Yenni RA et al. (2020) examines the non-structural Preparedness of the hospital in the face of disasters based on the hospital safety index. This research is a mixed study with a sequential explanatory design. The non-structural Preparedness of office equipment, furniture, and storage is 0.84% based on the hospital safety index. The results show that the obstacles hospitals face in implementing disaster preparedness are budget credits and the hospital’s focus on promoting non-structural Preparedness [75]. In the study of Parchami M and colleagues (2020) in the investigation of the safety situation and Preparedness of the hospitals of Ilam City against disasters, they admitted that in terms of office equipment in all the study hospitals, all the office equipment, including shelves, computers, and office furniture, are in a terrible condition and case of an accident, they will be destroyed. They failed, and no strengthening was done in any part of the hospital [13]. It was done in the study of MohammadiYegane et al. (2011) titled Assessment of Qualitative and Quantitative Performance of Equipment and the Non-structural Vulnerability of Tehran’s Elected Public Hospitals during an Earthquake. The safety level of hospital furniture was reported to be only 29% [54]. In a study conducted by Asefzadeh S and colleagues (2016), the safety score of office equipment and furniture and storage (fixed and movable) in Velayat Hospital and 50% in Rajaei Hospital has been obtained [39]. The study by Salari H and colleagues (2009) titled Preparedness of Governmental and Private Hospitals of Shiraz to Deal with Disasters was done—the lowest safety score related to office equipment and furniture (fixed and movable) [28]. In the study of Salari H et al. (2009), the lowest safety score was related to office equipment and furniture (fixed and movable) [28]. In the study of Sadeghi et al. (2015), in particular, Baath and 501 army hospitals had the highest safety percentage at 73%, and other army hospitals had the lowest safety percentage at 60% [45].

### Medical and laboratory equipment and supplies used for diagnosis and treatment

The studied hospital obtained a 58.82% safety level in medical supplies and equipment. Falling of nearby equipment and facilities on medical gases can cause irreparable critical and material damage. After the implementation of the FOCUS-PDCA model by the process owners and the implementation of corrective measures in this area, including measures such as installing a sterile device (CSSD) in the operating room and increasing the oxygen production capacity required in the areas of safety of medical supplies and equipment, the safety level score It was upgraded to 65%. In the study of Abd Rahman NH et al. (2023), 36 medical supplies and equipment articles have been selected and reviewed. It concludes that the reliability of medical devices is categorized into three main areas: risk management, performance prediction, and maintenance maintenance. Most studies emphasized prioritization, failure and risk analysis, and performance prediction management systems using artificial intelligence should be enhanced to reduce the probability of failure. Age of equipment, type of equipment, performance, risk and safety, failures, rate of use, use of standard parts, maintenance schedule, and preventive repairs, availability of support, equipment installation location, weather conditions, calibration, error codes, failure frequency, the state of services, the measures taken, the failure and the history of the incident can have an impact on the level of preparedness of medical supplies and equipment in disasters. Scientific data on actions taken after failure, maintenance and repairs, cost, and a new predictive analysis model using artificial intelligence are expected to improve the current situation [76]. Mandić-Rajčević et al. (2023) have investigated various organizations that have attempted to develop assessment methods to identify and manage hospital disaster preparedness weaknesses. This article aimed to evaluate safety using the Hospital Safety Index (HSI), which has an overall safety index of 0.82%, with structural, non-structural, and disaster management safety indices of 0.95, 0.74, and 0.75, respectively, which indicates the possibility of its performance in disasters. HSI’s detailed case-by-case analysis highlights the necessary improvements in emergency water and power supply, telecommunications, and emergency medical equipment that rendered PHC inoperable during the 2014 floods. Most cases related to primary health care facilities such as hospitals were considered, except some cases in medical equipment, patient care, and support services. Fine-tuning the HSI to primary healthcare settings, its formal translation into different languages, and facilitating scoring and analysis can lead to a valid safety assessment tool in primary healthcare centers [73]. In a study conducted by Asefzadeh S (2016) et al., the safety score of medical supplies and equipment in Velayat Hospital is 55.55%, and Rajaei Hospital is 95% [39]. In the study of Sadeghi M et al. (2015), Baath Hospital and 501 had the highest safety percentage at 64%, and Ahoz Hospital, 578, with 49%, had the lowest safety percentage in medical supplies and equipment [45].

## Conclusion

In general, in the non-structural evaluation of the studied hospital, it did not have optimal safety in facing accidents and disasters in some areas. The present study showed that the application of Total Quality Management (TQM) and, primarily, its application tool, FOCUS-PDCA, is efficient and helpful in improving the non-structural safety of hospitals. Using action research in the health field in accidents and disasters can open blind spots in different aspects of preparedness and safety (structural, non-structural, and functional). As in the present study, with the implementation of this model, the selected hospital’s non-structural safety was improved from a moderate to a reasonable level. This result can be considered a practical model in other medical centers and hospitals.

## Data Availability

Data is provided within the manuscript.
